# Description and outcomes of an interprofessional communication training program for adult oncology clinicians

**DOI:** 10.1007/s00520-025-10129-0

**Published:** 2025-11-13

**Authors:** Betty R. Ferrell, Haley Buller, Judith Paice, Myra Glajchen, Trace Haythorn

**Affiliations:** 1 Division of Nursing Research and Education, City of Hope, 1500 E Duarte Road, Duarte, CA 91010 USA; 2 Division of Hematology-Oncology, Northwestern University Feinberg School of Medicine, Chicago, IL USA; 3 MJHS Health System, Albert Einstein College of Medicine, New York, NY USA; 4 Haythorn Coaching & Consulting, LLC, Decatur, GA USA

**Keywords:** Communication, Interdisciplinary education, Palliative care, Cancer education

## Abstract

**Purpose:**

Effective communication is essential for the delivery of quality cancer care; however, few clinicians receive formal communication training. There is a growing need for communication training tailored towards the interdisciplinary team’s role in patient-centered care and addressing communication across all aspects of cancer care. The goals of this National Cancer Institute–funded training program were to (1) identify the eight domains of quality palliative care applicable across all stages of cancer, (2) demonstrate skills in key clinical areas of communication through lab sessions, and (3) develop goals for implementing communication skills training in practice through process improvement, staff education, and clinical care.

**Methods:**

The Interprofessional Communication Curriculum (ICC) training program, a train-the-trainer course for US-based adult oncology clinicians, included a 3-day training course with 1-year follow-up for ongoing support and to assess impact. Measures included pre-course institutional assessment, participant goal implementation, course evaluations, and surveys to assess communication training efforts post-course.

**Results:**

Five training courses included 388 clinicians (nurses, social workers, and chaplains) in dyads from 38 US states. The project included 30% under-represented minority participants. The post-course results revealed participants trained an additional 9746 clinicians: 5872 nurses, 1276 social workers, 586 chaplains, 1128 physicians, and 884 others including students.

**Conclusion:**

The ICC training course resulted in increased effectiveness in communication practice across domains of care. ICC is an effective train-the-trainer program for interdisciplinary communication training.

## Background

Communication plays an important role in the delivery of quality care and is recognized as an essential clinical skill in improving the quality of life for patients [1–4]. The field of palliative care continues to recognize communication as a key principle and priority in caring for patients with serious illnesses [1–6]. Effective communication in clinical settings has led to increased patient satisfaction with information and support provided by clinicians and has led to improved assessments and conversations about patient fears and concerns [5–10]. In addition, effectively communicated information by the care team throughout the continuum of care has been shown to help patients and their families make informed choices and have effective conversations about their goals of care [11–14]. In oncology, there is a growing need for effective communication skills for the entire interdisciplinary healthcare team to better address a cancer prognosis and care options, conduct efficient family meetings, and engage in effective spiritual and cultural assessments [10–12, 14, 15].

Interdisciplinary education has been identified as a key feature in preparing an adequately trained and coordinated oncology workforce in communication [16–19]. Studies have suggested the need for communication training tailored towards the interdisciplinary team’s role in patient-centered cancer care and addressing communication across all aspects of care [16–19]. Communication skills training can prepare an oncology workforce proficient in communication and improve the quality of patient-centered care provided to patients and their families [16, 17, 20–23]. Nurses, social workers, and chaplains, specifically, have reported that they are often present with patients and families after a diagnosis of a serious illness is communicated [17–19, 24] and therefore have significant potential to integrate communication skills within their role and provide training to others at their home institutions [18, 19].

While interdisciplinary education has been shown to impact cancer care, institutions often struggle to support interdisciplinary education, particularly education targeted towards communication skills-building [17–19]. Additional barriers to enacting institutional-wide training include a lack of funding to initiate or maintain programs, the imbalance of professional clinical representation in existing communication training programs, and a lack of experience in training others [17–19]. In recognition of the importance of interprofessional communication, a curriculum was developed to provide communication skills training to healthcare oncology teams with the intent of making effective communication a quality improvement measure in cancer care. The Interprofessional Communication Curriculum (ICC) Project, a national train-the-trainer communication training program for oncology clinicians led by City of Hope (COH) investigators, was approved by the COH institutional review board in accordance with the Declaration of Helsinki. Participants provided written consent to participate in the project. The 5-year project spanned March 2020–March 2025 with one national course held in each project year. The purpose of this training project was to provide oncology clinician teams with communication skills training and to help prepare them to provide communication skills training at their home institutions. This article reports on the outcomes of the 5-year (2020–2025) National Cancer Institute (NCI)–funded training grant (1R25CA240111-01A1), the ICC training program.

## Project methods

The learning objectives of the training courses were as follows:

- Objective 1: Identify the eight domains of quality palliative care applicable across all stages of cancer.
- Objective 2: Demonstrate skills in key clinical areas of communication through lab sessions.
- Objective 3: Develop goals for implementing communication skills training in practice through process improvement, staff education, and clinical care.

## Course design

### Curriculum development and agenda

The agenda for the 3-day ICC training course is depicted in Table 1. The curriculum is based on the current evidence regarding interdisciplinary communication research, and for each domain of quality care [1, 25]. The course was developed and organized by the eight palliative care domains, as defined by the National Consensus Project (NCP) Guidelines for Quality Palliative Care [1]. The domains include Structure and Processes of Care; Physical Aspects of Care; Psychological and Psychiatric Aspects of Care; Social Aspects of Care; Spiritual, Religious, and Existential Aspects of Care; Cultural Aspects of Care; Care of the Patient Nearing the End of Life; and Ethical and Legal Aspects of Care. The general framework for the course was to provide didactic lectures by leaders and researchers in the three targeted disciplines of nursing, social work, and chaplaincy to summarize the key evidence–based content. Each didactic lecture included areas of communication applicable to palliative care in each of the eight domains. Following each didactic lecture, participants were divided into small groups of fifteen people or less to participate in lab sessions. The lab sessions were intended as a time for using experiential learning such as role play, viewing video clips of communication scenarios, followed by discussion, self-assessment tools, and other methods to reinforce the content. Table 1 also depicts descriptions of lab session activities. Both lectures and lab sessions emphasized the train-the-trainer format of the program and included a focus on how to use the ICC materials to teach others, as each participant was expected to share the curriculum with colleagues at their home settings. The course was designed with the intention to improve participants’ ability to incorporate communication skills training into their roles as oncology clinicians while preparing them to serve as clinical resources to assist colleagues in properly communicating common patient concerns.

**Table 1:** ICC course agenda

**Day 1**	
**Time**	**Module**
8:00–9:00	Welcome: NCP Guidelines as a Framework for Interdisciplinary Communication and Domain 1: Structure and Process of Care
9:00–10:00	Domain 2: Physical Aspects of Care
10:00–10:15	**BREAK**
10:15–11:15	Lab Session: Communication Skills for Domain 2* *Description: Discuss the use of communication skills in pain and symptom management assessment conversations demonstrated in viewed vignettes; engage in pain and symptom management assessment role-play activity
11:15–12:15	Domain 3: Psychological and Psychiatric Aspects of Care
12:15–1:15	**LUNCH**
1:15–2:15	Lab Session: Communication Skills for Domain 3* *Description: Discuss listening activities and the use of them in teaching others; discuss and complete role play activity focusing on active listening skills in various clinical settings
2:15–3:00	Domain 4: Social Aspects of Care
3:00–3:15	**BREAK**
3:15–4:00	Lab Session: Communication Skills for Domain 4* *Description: Discuss and complete role play activity focusing on accessing family members’/caregivers’ quality of life (QOL) with the QOL Model as a guide
**Day 2**	
**Time**	**Module**
8:00–8:15	Welcome
8:15–9:15	Domain 5: Spiritual, Religious and Existential Aspects of Care
9:15–10:00	Domain 6: Cultural Aspects of Care
10:00–10:15	**BREAK**
10:15–11:15	Lab Session: Communication Skills for Domain 5 and Domain 6* *Description: Discuss and complete spiritual assessment conversations role-play activity; discuss and practice use of COAL (curiosity, openness, acceptance, love) in cultural assessment conversations
11:15–12:00	Domain 7: Care of the Patient Nearing the End of Life
12:00–1:00	**LUNCH**
1:00–1:45	Lab Session: Communication Skills for Domain 7* *Description: Discuss and complete reflective journaling activity focusing on EOL goals and providing patients with a “good death”; discuss preparing a family meeting and practice engaging in a family meeting
1:45–2:30	Domain 8: Ethical and Legal Aspects of Care
2:30–2:45	**BREAK**
2:45–3:45	Responsible Conduct of Research
3:45–4:30	Quality Improvement Strategies and Measuring Outcomes of Improved Communication
**Day 3**	
**Time**	**Module**
8:00–8:15	Welcome
8:15–9:00	Implementation of Communication Skills into Practice
9:00–10:00	Lab Session: Implementation of Communication Skills into Practice* *Description: Discuss challenges participants have faced in coaching frontline clinicians in engaging in difficult conversations; roleplay helping a frontline clinician navigate a roadblock in a prognosis and goals of care conversation
10:00–10:15	**BREAK**
10:15–11:15	Presentation of Team Goals
11:15–11:45	Summary, Evaluations, and Next Steps

### Participant criteria/application

The course was limited to US participants and was designed for in-person training; however, the first of the five courses was converted to virtual format due to COVID-19 travel restrictions. Participants were selected in a competitive process and recruited from social work, nursing, and chaplaincy departments in cancer centers across the USA. To qualify, participants were required to work in adult oncology settings and needed to apply in interprofessional dyads (e.g., nurse and chaplain, chaplain and social worker, or nurse and social worker). Recruitment was limited to the three disciplines due to funder feedback and pilot course data suggesting there were less training opportunities available for these key oncology professionals [17, 18].

The course application collected demographic information on applicants and their institutions. They were also asked to report their institution’s greatest challenges to improving communication. A letter of support was required from each participant’s immediate supervisor expressing support for their participation in the course, their completion of the post-course surveys, and the execution of their team goals after the training. Applications were reviewed by the project investigators using a structured evaluation tool. Participants who were selected received free course registration, a travel stipend that covered lodging costs during the course, course materials (e.g., textbook, curriculum, all lecture, and lab materials to use to teach others), and access to 12 months of the self-guided ICC Online learning modules via *Relias Learning* provides additional continuing education units and a certificate of completion. In addition, under-represented minority applicants could also apply for additional support to cover expenses related to travelling to an ICC course. These costs were paid through the NCI funding.

### Webinars

Monthly 1-h webinars were held for course participants for one year after course participation. Ten webinars were held over 12 months for each cohort with no webinars during August and December to avoid holidays and vacation schedules. These were designed to reinforce course content, provide new content as requested in post-course evaluations, share goal achievement by teams, and facilitate networking. Each webinar devoted 45 min to continued education and 15 min to open discussion related to the webinar presentation content or on team goal progression and experiences. Each webinar was offered twice to accommodate the range in time zones of participants. Example topics of the webinars included Loss, Grief, and Bereavement, Pain Management During the COVID-19 Pandemic, Spiritual Care Communication, and LGBTQ Conversations in Palliative Care. The monthly webinars averaged 85–90% participation rates.

## Outcome measurements

### Pre- and post-course self-rated effectiveness in communication

Each participant completed a pre-course assessment survey during the application process, and again at 6 and 12 months post-course, to rate their own perceived effectiveness in their communication skills across the eight domains using a Likert scale. In addition, participants were asked to provide the frequency with which they applied the learning in palliative care conversations (e.g., have a goals of care conversation with a patient, participate in a family meeting, or communicate with patients about spiritual care). Participants also reported on their implementation activities and how many colleagues they had taught using the course materials.

### Course evaluation

Participants completed daily course evaluations to evaluate the curriculum content and teaching methods and rate the clarity of presentations and the quality, value, and usefulness of content in the course. Participants also evaluated the value of the lectures and lab sessions to their clinical practice by rating the speaker’s knowledge/mastery of the topic, clarity and content quality, usefulness, and the appropriateness of teaching methods for each lecture and lab session. All evaluation data (pre- and post-course) were completed online by course participants on REDCap. Data were analyzed using descriptive statistics.

### Goal evaluation

During the application process, each team developed three goals for institutional change highlighting their plans for post-course training and for integrating communication skills into their practice. Throughout the 3-day course, teams were given the opportunity to share, discuss, and refine their goals. The 6- and 12-month follow-up reported teams’ efforts in providing communication skills training to others in their home institutions and enacting institutional-wide system changes in relation to communication.

## Results

### Demographic data

Three hundred eighty-eight participants from 38 states and Washington, DC, participated in an ICC training. Participants were mostly female (89%) and white (70%). Roughly 30% of the participants identified as an under-represented minority including 13.4% as Hispanics and 12.6% as Black or African American. About half (49.7%) were nurses followed by social workers (34.4%), and chaplains (14.4%). A few other professionals were accepted on special request, and this included three physicians, one death doula, and one psychologist. Over one-third (35.6%) of the teams worked at a hospital/oncology unit with the remaining teams employed at NCI Designated Cancer Centers (21.1%), Ambulatory/Outpatient Cancer Care Centers (20.2%), Community Cancer Centers (9.6%), or others such as hospices (13.5%).

### Pre-course institutional assessment evaluation

During the application process, participants were asked to rate their colleagues’ attitudes towards communication on a scale of 1 = not effective to 5 = very effective. Participants rated how effective staff at their institution were at communicating with cancer patients (3.2), how supportive their administration would be in prioritizing effective communication in clinical care (4.0), and how receptive they believed their staff would be in improving communication with cancer patients (4.0). When asked how important communication was to cancer care on a scale of 1 = not important to 5 = very important, participants reported importance as high (4.9). In addition, participants reported insight into their institution’s greatest challenges to improving communication. Roughly 68% (*N* = 265) reported “health professionals’ avoidance of talking about death” as their greatest barrier to communication improvement efforts followed by 59% (*N* = 227) identified “lack of time for education,” as another significant challenge.

### Course evaluation data

Participants completed daily course evaluations and rated the course’s effectiveness on a scale of 1 = lowest to 5 = highest. Participants rated the course as meeting their expectations at 4.8 and usefulness to their practice or professional development at 4.8. When asked if the context was current and whether the instructional materials were suitable or useful, participants rated both at 4.7. Participants also rated the lectures and lab sessions on a scale of 1 = not effective to 5 = very effective. Participants rated Spiritual, Religious and Existential Aspects of Care (4.8) and Care of the Patient Nearing the End of Life (4.8) as the most effective lectures. In terms of lab sessions, participants found the combined Cultural and Spiritual, Religious, and Existential Aspects of Care lab as the most effective (4.7). All sessions were rated within an average range of 4.5 to 4.8.

### Pre- and post-course assessment evaluation

Participants completed pre- and post-course assessment tools during the application, and at the 6- and 12-month follow-ups (87% response rate), to rate how effective they were in communicating with patients at their institutions during key points in treatment (e.g., diagnosis, during treatment, survivorship, at recurrence, when facing the end of life, at time of death, and during bereavement) and in relation to the eight NCP domains of care. The participants reported that their communication effectiveness across the eight NCP domains had increased since attending the course. In addition, participants reported an overall increase in the frequency in which they engaged in palliative care conversations across the continuum of care. Table 2 highlights and compares the results of baseline (application) to the final follow-up (12 months). A 6-month follow-up was also included and served as a check-in on progress.

**Table 2:** Pre- and post-course assessment evaluation data

	Baseline application (***N*** = 388)	12 months post-course (***N*** = 332)
How frequently in the past month have you done the following? 1 = not often, 5 = very often		
Discuss with a patient their goals of care?	3.9	4.0
Participate in a family meeting discussing/identifying a patient’s goals of care?	3.3	3.5
Recommend a patient consider a palliative care consult?	3.2	3.5
Provide spiritual care to a patient?	2.8	3.4
Speak with a family member regarding bereavement services?	2.9	3.3
Support clinical staff for impending death of a patient?	3.3	3.5
How effective are you in communicating with patients at your institution? 1 = not effective, 5 = very effective		
At the time of diagnosis	3.3	4.1
During treatment	3.7	4.4
During survivorship	3.1	4.0
At recurrence	3.3	4.2
Facing end of life	3.6	4.4
At time of death	3.3	4.1
During bereavement	3.0	4.0
Rate your effectiveness in communicating in each of the 8 NCP guideline domains. 1 = not effective, 5 = very effective		
Domain 1: The Structure and Process of Care (e.g., engaging in interdisciplinary communication and communication regarding a patient's goals of care)	3.6	4.4
Domain 2: Communicating Physical Aspects of Care (e.g., assessing physical symptoms, communicating findings with team members, educating patients/family members)	3.4	4.3
Domain 3: Communicating Psychological and Psychiatric Aspects of Care (e.g., assessing and communicating various psychological issues such as emotional distress, anxiety and depression with patients and family members)	3.5	4.4
Domain 4: Communicating Social Aspects of Care (e.g., addressing environmental and social factors that affect patient and family quality of life, addressing patient and family areas of need)	3.6	4.4
Domain 5: Communicating Spiritual, Religious, and Existential Aspects of Care (e.g., patient and family spiritual beliefs are assessed and communicated to the interdisciplinary team)	3.1	4.1
Domain 6: Communicating Cultural Aspects of Care (e.g., assessing and communicating patient and family values, beliefs and traditions related to health and decision making)	3.2	4.2
Domain 7: Communicating Care of the Patient Nearing the End of Life (e.g., communicating with patients and families about signs and symptoms of approaching death, discussing end of life goals of care with patient and family)	3.4	4.2
Domain 8: Communicating Ethical and Legal Aspects of Care (e.g., ensuring that patient-expressed values, care preferences, spiritual beliefs and cultural beliefs are elicited and routinely reviewed and communicating to the interdisciplinary team)	3.2	4.1

The dyads submitted a total of 636 outcome goals. The 6- and 12-month follow-up revealed that 28% of goals (*n* = 176) were completed and 29% (*n* = 186) were in progress. For the goals that were reported as delayed in completion or not started at the 12-month follow-up, dyads reported changes in staff, high patient workload, and limited managerial support as the main barriers to completion. A qualitative data analysis of thematic coding was conducted on the goals. The goals were categorized into three themes: (1) Training Development Efforts, (2) Training and Education Implementation Efforts, and (3) Quality Improvement and System Changes. Table 3 provides examples of dyad goals per theme.

**Table 3:** Dyad goal themes and examples

Themes	Example goals
Training development efforts	“Develop a training booklet for new staff of the outpatient team that reviews supportive services available to new patients and how to access and refer to them. This booklet would have a draft completed within 3 months and be finalized after review with administration after 6 months.” “Develop a tool to assess individual staff members’ comfort level in communicating with patients and families regarding oncology care/palliative medicine. This tool would be appropriate for use with multidisciplinary team members with varied roles.” “Develop a “fast facts” educational pamphlet on the roles of the palliative care team and chaplain including when is appropriate to consult & how to consult.” “Create a collaborative interdisciplinary committee of six members from different departments in our cancer center whose mission is to improve communication processes within our organization. Develop the team within 3 months of returning from training. Then, within 6 months, the team will plan the most impactful strategy to utilize materials and skills brought back from the ICC that will have the most impact for our patients.” “Develop a comprehensive care team journal for providers to document the significant experiences encountered during the end-of-life journey, offering a vital outlet for processing emotions and preventing burnout. Created and implemented by August 2025.” “Create and implement a post discharge survey by end of year 2022, for patients who were referred to the outpatient Palliative Care clinic, to assess communication effectiveness between the Palliative Care clinic and patient, within a 90-day period.”
Training and education implementation efforts	“Implement a staff training for oncology team members where one time per month during our weekly meetings we will discuss one of the eight domains of palliative care while modeling the communication skills that we have worked to enhance during the training.” “Establish a new employee orientation training or workshop on using effective interprofessional communication; training would be included in new hire checklists across center with goal of completion within first 90 days of employment.” “Identify and train at least four interprofessional team members to become trainers in Goals of Care Conversation skills in the outpatient setting, ensuring ongoing training to enhance interprofessional communication and collaboration in oncology and palliative care.” “Administer needs assessment with Symptom Management Department medical providers to determine education gaps in specific domain areas. Plan focused trainings on identified areas and offer annually, with 1:1 or smaller group consultation available as needed. Survey after training to measure.” “Provide a training of the eight domains of quality Palliative Care to our graduate social work students working in oncology during the 2024-2025 academic year to enhance their skills.” “Introduce 1–2 new communication skills learned every -other- month at our cancer care committee meeting, that will enhance the way they serve our veteran population and family. Provide techniques that may work to reach our minority patients.”
Quality improvement and system changes	“Develop a ten-minute nursing in-service introducing key players of the supportive care team (Social work, spiritual care, child life, case management, and supportive medicine MD), their function, and how to appropriately screen patients for referral to supportive care.” “Implement the use of meaningful and therapeutic art-based legacy projects for patients and loved ones nearing end of life, fostering a safe and healing space. Program ideas generated and supplies purchased by August 2025.” “For the next year, implement a quarterly virtual newsletter to introduce care team members and improve ongoing connections to facilitate communication.” “Identify appropriate assessment tools to evaluate the quality of interdisciplinary communication amongst disciplines on the Palliative Care team.” “Support and mentor interdisciplinary team members with collaborative approaches to patient communication and care during patient rounds and case conferences.” “Introduce and evaluate the effectiveness of using an evidence-based tool to enhance nurses comfort level related to goals of care conversations (i.e., Conversation Project, Serious Illness Conversation Guide). Pilot the tool at one of our six cancer centers in 2021. Submit an abstract in 2021 to a national oncology conference related to our pilot program.”

Nearly half of the goals (49.2%) focused on training and education efforts. The 6- and 12-month follow-up results revealed participants provided training or education on ICC course materials to 9746 clinicians: 5872 nurses, 1276 social workers, 586 chaplains, 1128 physicians, and 884 others including administrative staff, students, and volunteers.

## Limitations

The project achieved its intended outcomes, as outlined in the “Results” section, despite facing implementation challenges during the height of the COVID-19 pandemic. The project included 388 participants representing 86% of the original goal of 450 participants. The greatest challenge of the project was related to the need to convert the in-person format to virtual for the first course due to COVID-19. In addition, the second and third courses faced some last-minute participation cancellations due to COVID-19 travel restrictions which resulted in decreased participant numbers overall, and in some cases, participants attending without their respective partners. Participants reported a higher staff turnover rate due to COVID-19 resulting in some participants leaving the institution and the remaining partner delaying or foregoing their original goals without a partner.

## Discussion

The NCI-supported ICC training program addresses a key aspect of quality cancer care, the use of communication across all domains of care. The course is a unique program that offers the tools to enact patient-centered communication in serious illness. This innovative communication skills training program focuses on professionals in three disciplines in oncology: nurses, social workers, and chaplains, who are critical in providing psychosocial support in cancer centers and who are models of the best care available for patients. In addition, the post-course follow-up, monthly webinars, and ongoing support post-course make ICC different from other continuing educational programs available.

Investigators recognize the high retention rate for ICC can be partly attributed to the financial and administrative support provided through the grant funding, but participant feedback and post-course follow-up demonstrate that ICC’s train-the-trainer format provides a cost-effective approach to communication training for institutions with limited financial resources or those in rural communities. This training program can serve as a model for other clinical areas beyond oncology looking to enhance communication skills among their healthcare teams. In addition, the post-course goal progress data (28% complete; 29% in-progress) demonstrates that effective goal completion can occur within 12 months of training, but many goals, especially the goals focusing on quality improvement projects, require a longer time for implementation.

Nurses, social workers, and chaplains are critical in providing physical, psychosocial, and spiritual support in cancer centers and can significantly benefit from the use of the ICC course materials and training to educate staff and to initiate quality improvement to extend effective communication to more patients. Training programs such as ICC are essential for preparing the interdisciplinary cancer team to become effective communicators with patients, families, and their interdisciplinary colleagues.

With the completion of the NCI-supported project, investigators recognize the need for further education efforts in communication for all interdisciplinary clinicians and not limited to nurses, social workers, or chaplains in oncology. Former participants of ICC provided verbal and written feedback expressing the wish to invite colleagues outside the three disciplines and colleagues in areas beyond oncology to receive this training. An adapted version of ICC will be offered at the End-of-Life Nursing Education Consortium (ELNEC) summits, which are national palliative care training courses that are hosted biannually in the USA. The adapted version of the curriculum will include all clinicians interested in communication training and will be expanded to all areas of serious illness care. More information on the continuation of interdisciplinary communication training is available at https://www.aacnnursing.org/elnec.

## Data Availability

No datasets were generated or analysed during the current study.
